# Enhancing water use efficiency and grain yield of wheat by optimizing irrigation supply in arid and semi-arid regions of Pakistan

**DOI:** 10.1016/j.sjbs.2021.10.018

**Published:** 2021-10-11

**Authors:** Mahwish Jabeen, Sajid Rashid Ahmed, Mukhtar Ahmed

**Affiliations:** a College of Earth and Environmental Sciences, University of the Punjab, Lahore, Pakistan; b Agriculture and Biological Engineering Department, University of Florida, Gainesville, USA; c PMAS, Arid Agriculture University, Rawalpindi, Pakistan; d Department of Agricultural Research for Northern Sweden, Swedish University of Agricultural Sciences, Umea, Sweden

**Keywords:** Crop modelling, Crop water productivity, Deficit irrigation, Irrigation management, Water sustainability

## Abstract

The lack of good irrigation practices and policy reforms in Pakistan triggers major threats to the water and food security of the country. In the future, irrigation will happen under the scarcity of water, as inadequate irrigation water becomes the requirement rather than the exception. The precise application of water with irrigation management is therefore needed. This research evaluated the wheat grain yield and water use efficiency (WUE) under limited irrigation practices in arid and semi-arid regions of Pakistan. DSSAT was used to simulate yield and assess alternative irrigation scheduling based on different levels of irrigation starting from the actual irrigation level up to 65% less irrigation. The findings demonstrated that different levels of irrigation had substantial effects on wheat grain yield and total water consumption. After comparing the different irrigation levels, the high amount of actual irrigation level in semi-arid sites decreased the WUE and wheat grain yield. However, the arid site (Site-1) showed the highest wheat grain yield 2394 kg ha^−1^ and WUE 5.9 kg^−3^ on actual irrigation (T_1_), and with the reduction of water, wheat grain yield decreased continuously. The optimal irrigation level was attained on semi-arid (site-2) with 50% (T_11_) less water where the wheat grain yield and WUE were 1925 kg ha^−1^ and 4.47 kg^−3^ respectively. The best irrigation level was acquired with 40% less water (T_9_) on semi-arid (site-3), where wheat grain yield and WUE were 1925 kg ha^−1^ and 4.57 kg^−3^, respectively. The results demonstrated that reducing the irrigation levels could promote the growth of wheat, resulting in an improved WUE. In crux, significant potential for further improving the efficiency of agricultural water usage in the region relies on effective soil moisture management and efficient use of water.

## 1 Introduction

Providing sufficient water for crop production and food insecurity are Pakistan’s major challenges in the 21st century. Unsustainable use of fresh water in the agriculture sector can hamper crop productivity and food security (Yang et al., 2015, Li et al., 2019b, Xu et al., 2020). Pakistan is one of the world's most arid countries with an average yearly precipitation of approximately 250 mm (UNEP, 2011) – uses over 94% of water withdrawal in agriculture (FAO, 2016b). A capacity of about 18 MAF of water from hill torrents has been estimated. The overall irrigation benefits even 12 times the direct, onsite benefits if all identifiable social and economic are taken into account (World Bank, 2016). Pakistan is not utilizing its natural water resources efficiently with the adoption of modern irrigation (i.e. drip and sprinkler) strategies (Ahmad et al., 2015). It would be difficult to enhance global food insecurity unless the use of agricultural water supplies is sustainable (Biswas, 2008, García, 2008).

The scarcity of water is the major limiting factor for the production of crop in arid and semi-arid regions (Deng et al., 2006, Piao et al., 2010). Presently, irrigation remains an essential method of ensuring the production of food, with 40% of world crops cultivated on irrigated land (Ertek and Yilmaz, 2014). Water usage is expected to rise 50% after 30 years, with an estimated 4 billion people – one-half of the world's population – living in acute water shortage by 2030, primarily in Africa, the Middle East, and South Asia (Bos et al., 2005). Thus, the improvement of agricultural production and WUE in arid and semi-arid regions is a significant challenge. The warmer condition leads to increased agricultural drought throughout the western side of Punjab (Qamar et al., 2018). (Yasin et al., 2001) reported that irrigation drainage water is 54% of the total water drainage for agricultural production in the Dera Gazi Khan.

Application of water that is below the required evapotranspiration level is deficit irrigation (Fereres and Evans, 2006). Under the deficit irrigation to meet the maximum evapotranspiration rate the supply of water is relatively decreased (English, 2015). Based on soil water availability at the root crop zone, automated irrigation systems schedules the irrigation applications on a real-time basis and increase water use efficiency (WUE) by saving a substantial amount of water (Ojha et al., 2015). However, deficit irrigation is a simple technique for the improvement of economic output under limited water supply, and it also imposes many adjustments in the agriculture system under the reduced water supply (Molden et al., 2007).

Production of more food with less water is only accomplished with better agronomic management strategies considering depleting aquifer conditions. Improving agricultural productivity and consumption of water has been taken into account as agricultural management of water (Molden et al., 2003, Bessembinder et al., 2005). Crop simulation models should be applied to enhance and implement a better strategy to improve crop output under different irrigation systems. Crop production functions are also efficient ways of estimating yields through transpiration (T) or crop evapotranspiration (ET) and yield relations because they significantly influenced water usage. The function of crop production is the ratio of water consumed during ET and yield production. Many researchers have suggested a linear link to a soil water deficit between wheat (*Triticum aestivum* L.) production and ET (Doorenbos et al., 1979, Zhang et al., 1998, Aiken et al., 2013). The water requirement for a crop is evaluated from standard ET equations in the irrigation method based on ET (Jabloun and Sahli, 2008).

Decision support system for agro-technology transfer (DSSAT) is an extensive decision system that facilitates to easily generate databases for weather, soil, experimental data for the implementation and long-term validation of single-season and sequenced crop systems based on management, genetic, climate variables (Jones et al., 2003). The DSSAT can effectively evaluate water management scenarios to facilitate better recommendations for improved water management. DSSAT seasonal module was used to analyse the best treatment for the maximum and stable production of wheat under efficient irrigation practices (Jones et al., 2003). Crop development and growth are core aspects of the DSSAT as the model simulates crop growth and yield (Galindo et al., 2018).

There is a dire need to be worked on water balance and improve wheat grain yield by using simulation modelling and arid and semi-arid conditions of Dera Ghazi Khan Pakistan. The research was carried out to assess the optimal utilization of irrigation water and conserved water for other purposes. The specific objective of this study was, the assessment of wheat grain yield and WUE under limited irrigation practices in the arid and semi-arid region of Pakistan.

## 2 Materials and methods

### 2.1 Study site description

**Fig. 1:**
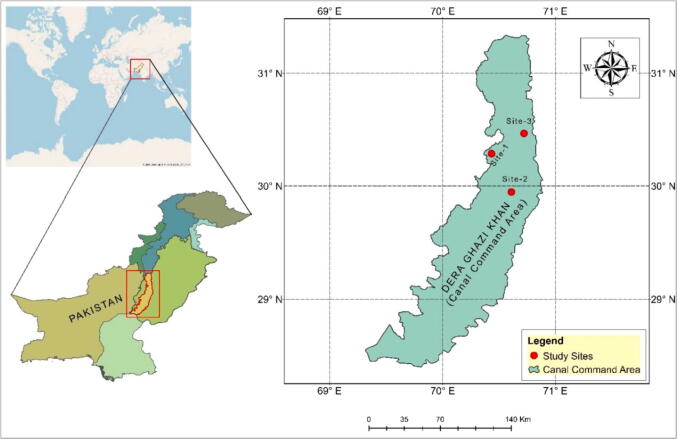
Canal command areas of Dera Ghazi Khan (Study Area).

The study was conducted by using baseline (1981–2010) for canal command areas of Dera Ghazi Khan (30.0489° N, 70.6455° E), Punjab, Pakistan (Fig. 1). The study area is part of alluvial plains at the west of the Indus River and extends towards the foothills and uplands of the Sulaiman Mountains where irrigation is mandatory with the seasonal rainfall for sustainable crop production. The total area of Dera Ghazi Khan is 11294 km^2^. Dera Ghazi khan district has four different seasons with both arid and semi-arid climates. Taunsa Barrage on the Indus River is the main source of irrigation in Dera Ghazi Khan District. The average temperature and mean monthly sunshine hours during the period between 1981 and 2010 were 24.2 °C and 2987 respectively. The average annual precipitation for the long term (1981–2010) was 268.8 mm and 221.5 mm, respectively.

**Fig. 2:**
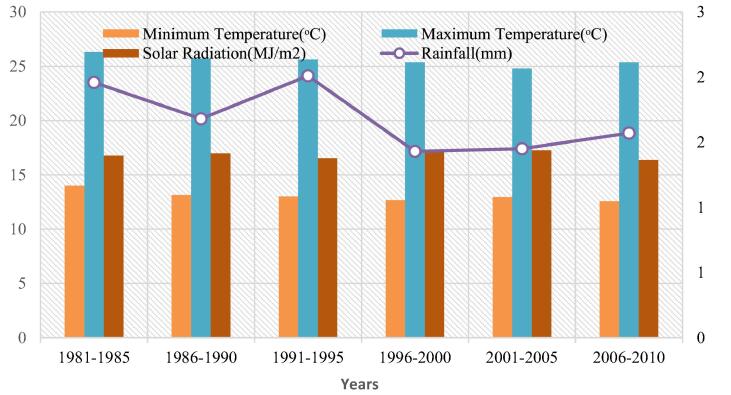
Climatic condition of site-1.

**Fig. 3:**
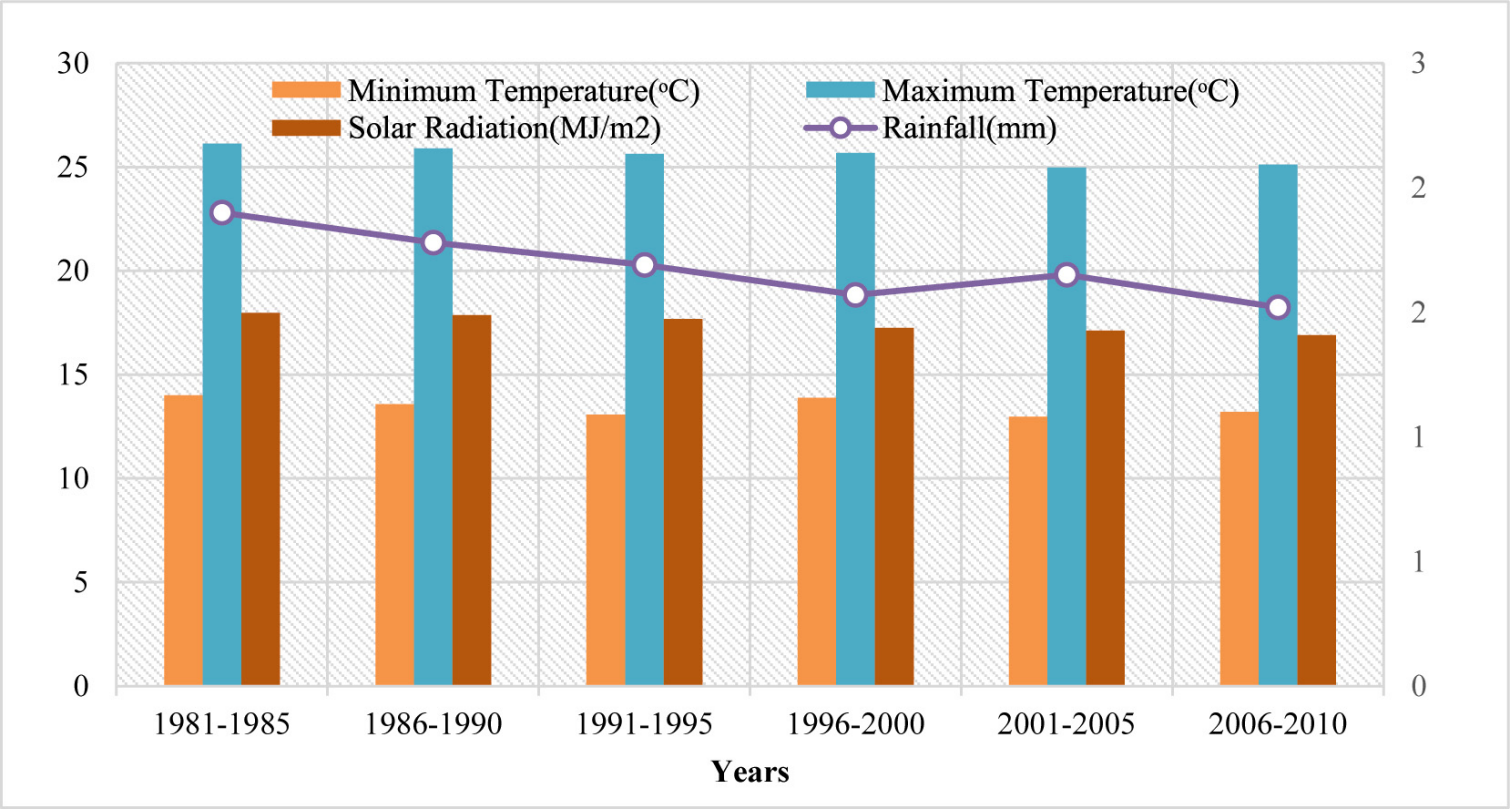
Climatic condition of site-2.

**Fig. 4:**
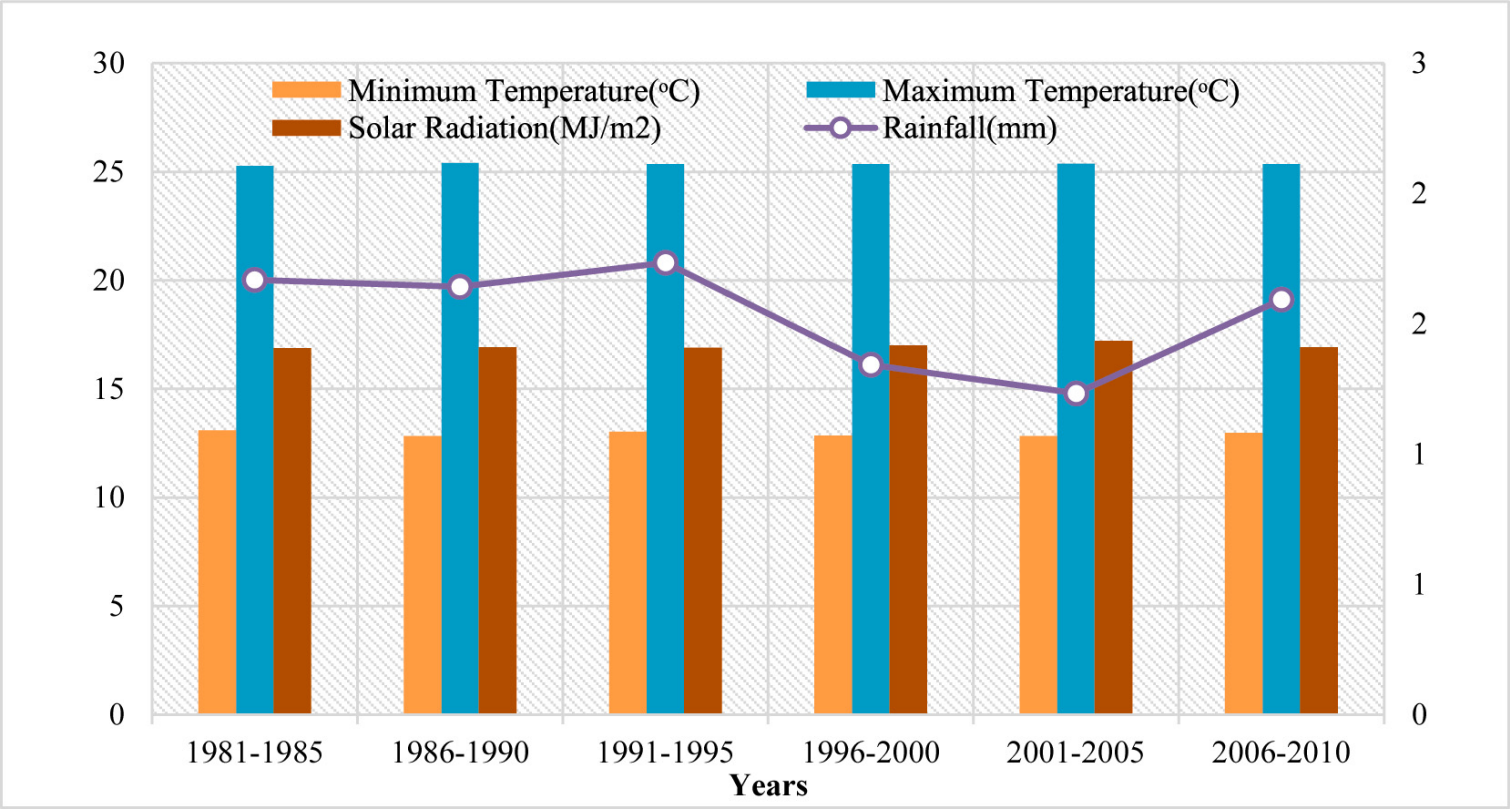
Climatic condition of site-3.

### 2.2 Model inputs

The long-term (1981–2010) baseline observation data that included minimum and maximum temperatures, daily precipitation (Fig. 2, Fig. 3 and 4) for three study sites were obtained from the Pakistan Meteorological Department (PMD). Solar radiation data were collected for the study area from a public domain online source the Global Land Data Assimilation System (GLDAS). The DSSAT model is based on the daily precipitation (mm), solar radiation (MJ/m^2^), maximum and minimum temperature (°C) for all sites of the study area. Soil profile information was acquired from the Soil Survey of Pakistan (Table 1). Actual practices followed in the field, crop management data, and irrigation water used as input to the DSSAT model that provided to each treatment.

**Table 1:** Description of irrigation treatments.

**Treatment Name**	**Treatment Description**	**Treatment Name**	**Treatment Description**
T_1_	Actual Irrigation (255 mm)	T_8_	35% Less (166 mm)
T_2_	5% Less (242 mm)	T_9_	40% Less (153 mm)
T_3_	10% Less (229 mm)	T_10_	45% Less (140 mm)
T_4_	15% Less (217 mm)	T_11_	50% Less (128 mm)
T_5_	20% Less (204 mm)	T_12_	55% Less (115 mm)
T_6_	25% Less (191 mm)	T_13_	60% Less (102 mm)
T_7_	30% Less (178 mm)	T_14_	65% Less (89 mm)

**Table 2:** Soil data used in DSSAT Simulations.

**SITE-1**												
**Soil Parameters**												
**Depth(cm)**	**SLCL(%)**	**SLCI (%)**	**SLOC (%)**	**SLHW**	**SCEC (cmol(^+^) kg^−1^)**	**SLNI (%)**	**SLLL (cm^3^ cm^−3^)**	**SDUL (cm^3^cm^−3^)**	**SSAT (cm^3^cm^−3^)**	**SBDM (g cm^3^)**	**SSKS (cm h^−1^)**	**SRGF**
**10**	12	35	0.3	8.5	12.1	0.03	0.066	0.184	0.43	1.42	7.14	1
**45**	8	31	0.22	8.5	12.3	0.02	0.089	0.213	0.426	1.43	4.13	0.58
**75**	6	15	0.09	8.8	13.9	0.01	0.034	0.133	0.388	1.54	14.08	0.3
**100**	10	33	0.09	8.9	13.9	0.01	0.109	0.219	0.414	1.47	3.36	0.17
**130**	18	30	0.3	7.6	13.9	0.03	0.137	0.29	0.43	1.47	1.09	0.25

**SITE-2**												
**3**	16	42	0.37	7.4	9.9	0.06	0.102	0.243	0.452	1.38	2.9	1
**18**	26	56	0.27	7.3	12.1	0.04	0.233	0.348	0.497	1.26	0.79	1
**40**	27	52	0.19	7.1	12.5	0.02	0.134	0.295	0.504	1.27	1.81	0.56
**60**	28	40	0.24	7.4	12.5	0.02	0.149	0.271	0.458	1.39	1.73	0.37
**80**	27	30	0.3	7.6	13.9	0.03	0.137	0.29	0.43	1.47	1.09	0.25
**95**	26	33	0.2	7.6	13.9	0.02	0.135	0.281	0.424	1.46	1.08	0.17

**SITE-3**												
**9**	21	24	1.07	8.9	9.3	0.13	0.121	0.237	0.437	1.46	2.77	1
**30**	38	26	0.62	8.9	9.3	0.11	0.216	0.339	0.452	1.52	0.43	0.68
**53**	33	29	0.43	8.9	9	0.09	0.197	0.304	0.443	1.53	0.79	0.44
**92**	33	22	0.23	7.95	7.7	0.06	0.178	0.286	0.396	1.55	0.74	0.23
**130**	31	22	0.16	9	5.9	0.05	0.228	0.309	0.399	1.53	0.4	0.11

SLCL = clay percentage; SLCI = silt percentage; SLOC = organic carbon; SLHW = pH by water extraction; SCEC = cation exchange capacity; SLNI = total nitrogen concentration; SLLL = lower limit of plant extractable soil water; SDUL = drained upper limit; SSAT = saturated upper limit; SBDM = bulk density; SSKS = saturated hydraulic conductivity; SRGF = root growth factor.

### 2.3 Comparison of irrigation scenario

After the successful evaluation of the DSSAT model, this process-based method comes to find the optimal irrigation water use strategy. The fourteen irrigation scenarios (T_1_-T_14_) representing various irrigation levels were developed by reducing the irrigation water application amount and holding irrigation timings constant for each irrigation event (Table 2). Irrigation scenarios comparison made for the baseline (1981–2010). Long-term simulations run by using the weather and experimental datasets. The trend of wheat grain yield to each scenario was estimated and determined whether the yield had reached a stable maximum value and then decline started (Table 1).

Simulation scenarios were developed to signify the consistent initial environmental conditions, actual irrigation schedules were used in the study. The irrigation water use efficiency (IWUE) was evaluated (Equation (1)) for each treatment in three study sites where IWUE is in kg m^−3^. Equation (1) is the best fit for various experimental conditions in the study area (Howell et al., 2004). Comparisons were performed to a baseline scenario that applied the same initial condition as the other simulations in the testing range.

(1) $$\mathrm{IWUE}=\frac{Y}{\mathrm{ET}}$$

Where:

Y = Grain Yield

ET = Evapotranspiration (mm)

The following equation was used to estimate the transpiration factor:

(2) $$\mathrm{TranspirationFactor}=\frac{\mathrm{ET}}{\mathrm{EP}}$$

Where:

ET = Evapotranspiration (mm)

EP = Potential Evapotranspiration (mm)

### 2.4 Model description

The soil water balance model of DSSAT v4.75 was used in this research. This one-dimensional model measures soil water content changes due to rainfall infiltration and irrigation on regular basis. The model has used the “Tipping Bucket” method for measuring the soil water drainage when the content of the soil layer is above the drained upper limit (Ritchie, 1998). An assessment of soil water diffusivity and variations in the water holding capacity of adjacent layers was used to derive upwards unsaturated flow (Ritchie, 1998). Daytime infiltration of soil water was determined by subtracting surface runoff from rainfall that occurred on the same day. The soil Conservation method is adopted to segregate the precipitation into infiltration and run-off that is based on a ‘curve number’ which tends to account for slope, tillage, and texture of the soil. (Saxton et al., 1986) developed by the modified method used soils layers and soil water after rainfall occurs.

Water drainage was assessed by the soil profile where drainage parameter in soil depth is considered to be constant. Saturated hydraulic conductivity is associated with the amount of water filtered through any soil layer. The model can simulate poorly drained soils and water tables by use of this feature. The soil water of each layer is adjusted by subtracting or adding daily water inflows.

The DSSAT water module can be shown as follows:

(3) ΔS=P+I-T-E-R-D

where: P as a precipitation, I as an irrigation, T as a plant transpiration, E as a soil evaporation, R as a runoff, and D as a drainage.

This procedure has a direct impact on the water content of the soil profile. DSSAT requires soil water content data, the upper limit drainage, and saturation to determine processes such as root uptake, drainage, and soil evaporation (DeJonge et al., 2012).

### 2.5 Agronomic and crop management

The local wheat cultivar (Sahar-2006) was planted between November 1 and November 15. Seeds were planted in plots 5 rows of 14 m in length and 0.75 cm in furrows at 0.07 m depth. The wheat crop was harvested between March 30 and April 15. Plots received 120 kg ha^−1^ nitrogen as urea and 250 kg ha^−1^ phosphorus as single super phosphate before planting based on the recommended dose. Fertilization of soil is a significant factor of crop management in ensuring plant growth with adequate nutrients (according to soil analysis). 70% of urea was applied at 19 and 20 days after planting (DAP) and the remaining was applied at 51 and 60 DAP. There were four irrigation applications as 21, 51, 96, 126 DAP were applied to the study area. A reduction in water from every irrigation application determined the potential crop water requirement because this area receives more canal water that reduces output.

### 2.6 Model calibration

DSSAT calibration is the adjustment of functions and parameters so that simulated data is the same or very close to data obtained from the experimental field. DSSAT model has been validated by a holdout cross-validation. The simulated results substantiates sufficient accuracy with the observed wheat grain yield recorded. The DSSAT simulated yield for specific site shows 2393 kg ha^−1^, 1630 kg ha^−1^, and 1815 kg ha^−1^ for site 1, 2 and 3 respectively. However, the observed values 2354 kg ha^−1^, 1645 kg ha^−1^, 1805 kg ha^−1^ for site 1, 2 and 3 respectively. The observed and simulated values shows close resemblance representing the fitness of the designed methodology.

## 3 Results

### 3.1 Effects of deficit irrigation on wheat grain yield

**Fig. 5:**
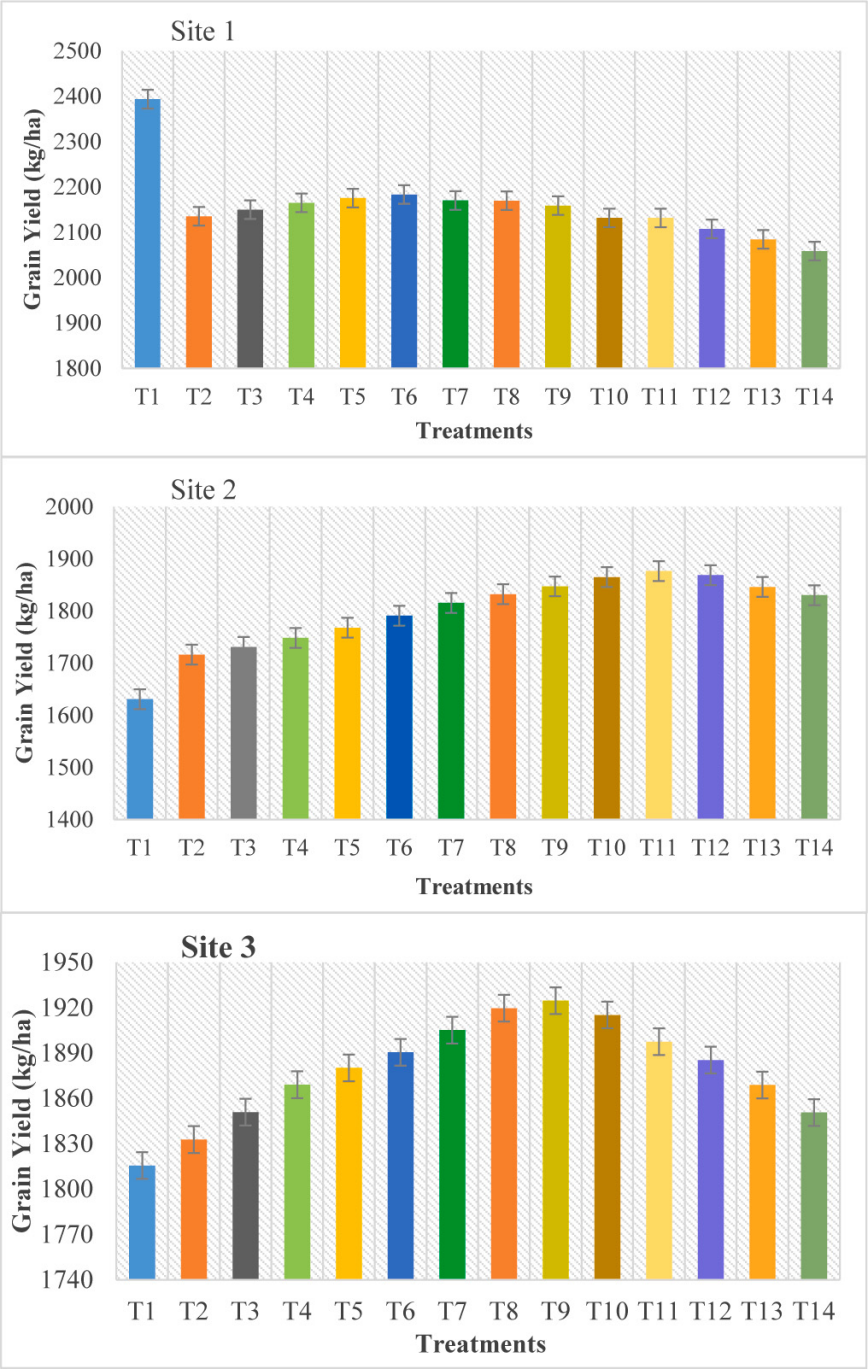
**Effects of deficit irrigation on grain yield of wheat.** T_1_ = 255 mm; T_2_ = 242 mm; T_3_ = 229 mm; T_4_ = 217 mm; T_5_ = 204 mm; T_6_ = 191 mm; T_7_ = 178 mm; T_8_ = 166 mm; T_9_ = 153 mm; T_10_ = 140 mm; T_11_ = 128 mm; T_12_ = 115 mm; T_13_ = 102 mm; T_14_ = 89 mm.

The real irrigation dates and depth applied by the farmers were obtained from the Irrigation Department of Pakistan and compare with the optimal irrigation scenarios for the best WUE. The study area receives more water than required for a wheat crop where two sites show the increasing trend while reduction in the irrigation so there’s more potential of crop yield with water management in this area. The optimal irrigation scenario was acquired by adjusting the irrigation practices to fulfil the need of crop water requirement with the output of maximum yield. Fourteen treatments (T_1_-T_14_) were used to simulate wheat grain yield to identify the best irrigation practice that efficiently used irrigation water and give high yield with less water (Table 1). 14 treatments have been started with actual irrigation water and then up to 65% less water utilized amongst all treatments with a 5% difference – the lowest wheat grain yield has been observed in site-2 relative to irrigated water levels and other sites (Table 3). Wheat grain yield of all three sites by using different irrigation practices changed significantly (Fig. 5).

**Table 3:** The effects of different irrigation levels on the water use efficiency and grain yield of wheat.

**SITE-1**														
**Treatments**	**T_1_**	**T_2_**	**T_3_**	**T_4_**	**T_5_**	**T_6_**	**T_7_**	**T_8_**	**T_9_**	**T_10_**	**T_11_**	**T_12_**	**T_13_**	**T_14_**
Yield (kg ha^−1^)	2394	2135	2150	2165	2176	2184	2170	2170	2159	2132	2127	2108	2085	2059
ET (mm)	400.6	423.4	423	422.7	422.3	422.1	421.6	421.3	420.8	420.5	419.2	408.2	400.3	388.5
WUE (kg^−3^)	5.975	5.043	5.083	5.122	5.152	5.173	5.148	5.150	5.130	5.069	5.085	5.163	5.207	5.299

**SITE-2**														
Yield (kg ha^−1^)	1631	1716	1731	1749	1768	1791	1816	1832	1847	1865	1877	1869	1846	1830
ET (mm)	400.6	423.4	422.7	422.5	422.3	422.1	421.6	421.6	419.3	418.5	410.2	402.8	397.8	382.7
WUE (kg^−3^)	4.071	4.054	4.092	4.137	4.186	4.243	4.306	4.349	4.390	4.435	4.477	4.578	4.612	4.711

**SITE-3**														
Yield (kg ha^−1^)	1815	1833	1821	1869	1880	1890	1905	1920	1925	1915	1896	1885	1867	1851
ET (mm)	400.6	423.4	422.7	422.5	422.3	422.1	421.6	421.6	419.3	418.5	410.2	402.8	397.8	382.7
WUE (kg^−3^)	4.53	4.33	4.38	4.42	4.45	4.48	4.52	4.56	4.57	4.55	4.53	4.62	4.67	4.76

The irrigation amounts under different irrigation levels are T_1_ = 255 mm; T_2_ = 242 mm; T_3_ = 229 mm; T_4_ = 217 mm; T_5_ = 204 mm; T_6_ = 191 mm; T_7_ = 178 mm; T_8_ = 166 mm; T_9_ = 153 mm; T_10_ = 140 mm; T_11_ = 128 mm; T_12_ = 115 mm; T_13_ = 102 mm; T_14_ = 89 mm; mm = millimetre; WUE = water use efficiency.

Water productivity (WP) was changed significantly due to the differences in wheat grain yield. Site-1 had the highest wheat grain yield on actual irrigation that is 2394 kg ha^−1^ and yield decreased with decreasing irrigation level (Table 3). Site-1 has clay loam properties and with a 10% decrease in actual irrigation yield decreases up to 2135 kg ha^−1^. Site-1 has a higher wheat grain yield on actual irrigation treatment.

Actual irrigation on site-2 produced a wheat grain yield of 1631 kg ha^−1^. But with the decrease of water, wheat grain yield increases gradually with up to 50% less water, and after that yield shows the decreasing trend. The highest grain yield of wheat on Site-2 with a 50% reduction in irrigation water was 1877 kg ha^−1^. Site-3 also shows an increasing yield trend with a reduction of irrigation application. Where 40% less water reduction showed the highest yield that is 1925 kg ha^−1^ and after that yield gradually reduced with the decreased irrigation.

### 3.2 Effects of evapotranspiration (ET) on wheat grain yield

**Fig. 6:**
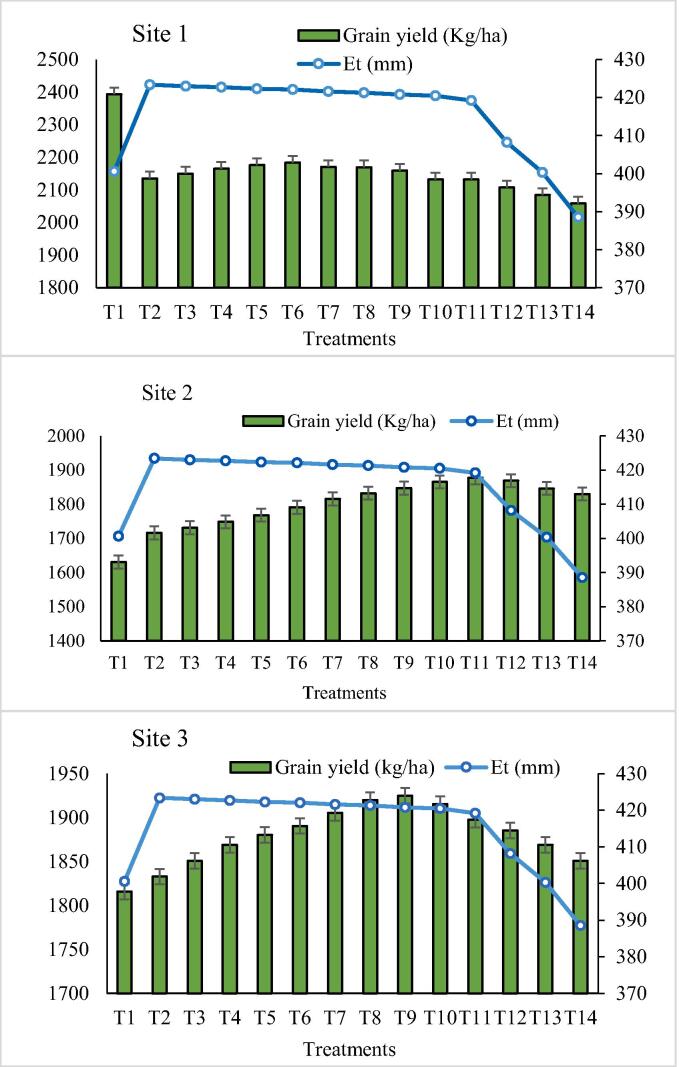
**Comparison of evapotranspiration (ET) and wheat grain yield on different irrigation levels** T_1_ = 255 mm; T_2_ = 242 mm; T_3_ = 229 mm; T_4_ = 217 mm; T_5_ = 204 mm; T_6_ = 191 mm; T_7_ = 178 mm; T_8_ = 166 mm; T_9_ = 153 mm; T_10_ = 140 mm; T_11_ = 128 mm; T_12_ = 115 mm; T_13_ = 102 mm; T_14_ = 89 mm.

The evapotranspiration trend for semi-arid sites (2 and 3) was almost same where the yield changed due to temperature and other biophysical factors (Fig. 6). Site-1 is an arid region where the low evapotranspiration showed a higher yield. The actual water supply was 5% and 10% fewer irrigation levels specify 423 mm highest ET rate and least ET 388 mm was observed on T_14_. Maximum wheat grain yield 2393 kg ha^−1^ was recorded on ET level 400.6 mm. The highest evapotranspiration 423 mm was observed at T_2_ and the least evapotranspiration 382 mm was recorded at T_14_ where the grain yield of wheat was 1716 kg ha^−1^ and 1876 kg ha^−1^, respectively on Site-2. The maximum value of ET on Site-3 has observed 423 mm and least ET 382 mm at 5% and 65% less irrigation treatment respectively, where grain yield of wheat was 1832 kg ha^−1^ at highest ET rate and 1850 kg ha^−1^ at lowest ET.

### 3.3 Effects of irrigation levels and water use efficiency on wheat grain yield

By improving the irrigation interval and level of irrigation, the values of wheat grain yield and WUE showed an increasing trend. Site-1 received adequate water and the deficient water application decreased wheat grain yield, but site-2 and 3 are irrigated areas and received more water than required however the wheat grain yield increased while a decrease in water in these two sites.

**Fig. 7:**
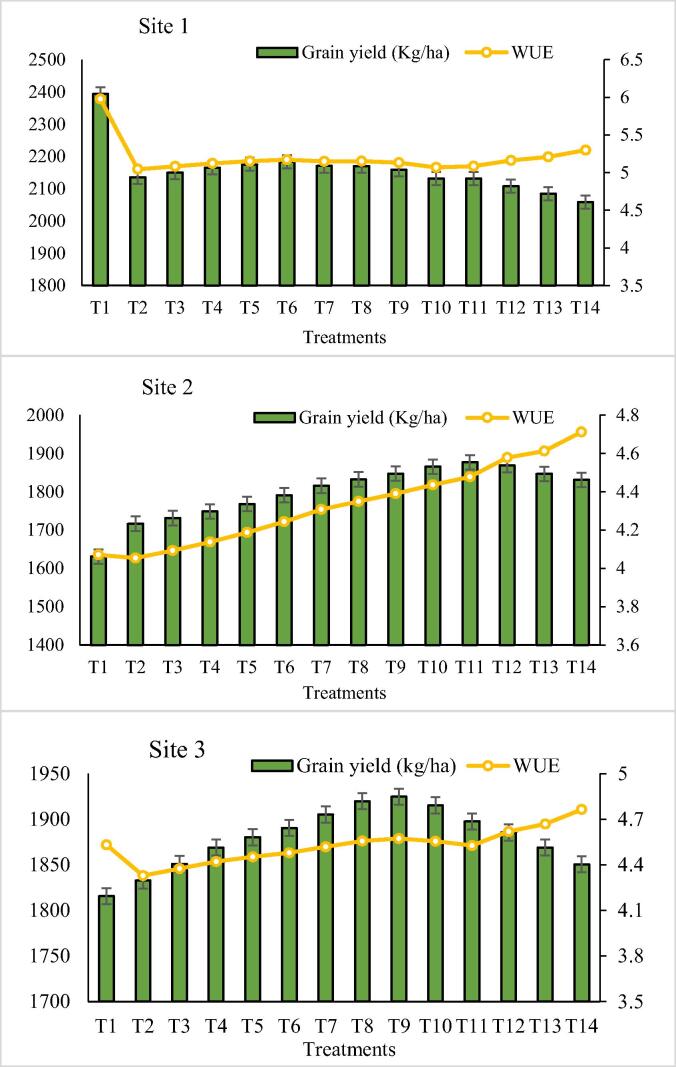
**Comparison of water use efficiency and wheat grain yield on different irrigation levels.** T_1_ = 255 mm; T_2_ = 242 mm; T_3_ = 229 mm; T_4_ = 217 mm; T_5_ = 204 mm; T_6_ = 191 mm; T_7_ = 178 mm; T_8_ = 166 mm; T_9_ = 153 mm; T_10_ = 140 mm; T_11_ = 128 mm; T_12_ = 115 mm; T_13_ = 102 mm; T_14_ = 89 mm.

Water use efficiency was decreased due to a decrease in irrigation levels and wheat grain yield also showed a declining trend at Site-1 (Fig. 7). The highest WUE was recorded 5.9 kg^−3^ on T_1_ where the yield was 2393 kg ha^−1^. T_14_ indicated the higher WUE 4.71 kg^−3^ at Site-2 where the simulated yield was 1846 kg ha^−1^. Greater WUE 4.76 kg^−3^ was observed at T_14_ treatment where the wheat grain yield was 1850 kg ha^−1^ (Table 3).

## 4 Discussion

Water use efficiency and crop yields in arid and semi-arid areas of Punjab are strongly influenced by the irrigation application rate substantially. However, excessive irrigation application reduced the crop production of wheat and also lower the WUE (Yang et al., 2015, Li et al., 2019b, Xu et al., 2020). The major water resource for the production of wheat is the available soil water in the growing season especially in arid and semi-arid areas (Hao et al., 2014).

The optimal irrigation scenario consisted of adjusting the irrigation practices to match the crop water requirement with the maximum output (i.e. wheat grain yield). It is important to emphasize that full irrigation treatment (T_1_) did not always result in the highest yield. This shows the different levels of irrigation had positive and negative impacts on wheat grain yield. This study examined the impact of irrigation supply on wheat grain yield and WUE applied on arid and semi-arid areas. The results indicated that the study area receives more water than is required for a wheat crop where two sites (semi-arid) showed the increasing trend while the reduction in irrigation so there’s more potential of crop yield with water management in this area (Wang, 2017).

Our findings also indicated that water scarcity has a negative impact on wheat grain yield in arid areas (i.e. Site 1 T_2_ and T_14_). Arid site (Site-1) has the highest yield on actual irrigation (T_1_) that was 2394 kg ha^−1^ and with the reduction of water wheat grain yield continuously decreased. However, after comparing the different irrigation levels, a high amount of actual irrigation level decreased the wheat grain yield and WUE in semi-arid sites (Site-2 and 3). There has been a substantial increase of WUE by the application of less water to wheat in semi-arid areas (Singh and Malik, 1983, Zhang et al., 2012, Thamer et al., 2019). Thus, the wheat crop requires moisture for its growth but in a certain capacity. We can save water by reducing the irrigation water level by 40% from the actual irrigation scenario by not sacrificing wheat grain yield. However, the harvest wheat grain yield is not only influenced by weather conditions but also human decision-making.

WUE values estimated in our study ranged from 4.05 to 5.975 kg^−3^ which were broadly in accordance with the results of (Jin et al., 2020), and their WUE range was 4.9–6.89 kg^−3^ for different treatments of deficit irrigation. The aforementioned study, nevertheless, did not incorporate a comparison of arid and semi-arid sites to optimize reduced irrigation as included in our study. WUE was 5.9 kg^−3^ also higher on the actual irrigation (T_1_) while compared with other 13 treatments. Optimal irrigation level obtained on site-2 with a decrease in 50% (T_11_) less water and the highest yield was recorded 1925 kg ha^−1^, whereas the WUE was 4.47 kg^−3^. Site-3 was also a semi-arid site, where the best irrigation level was acquired with 40% less water (T_9_), the wheat grain yield and WUE was 1925 kg ha^−1^, 4.57 kg^−3^ respectively. By reducing the irrigation levels could promote the growth of wheat resulting in the improved WUE in semi-arid regions (Xu et al., 2018). After evaluating the high WUE and wheat grain yield, T_1_ (Site-1), T_11_ (Site-2), and T_9_ (Site-3) were considered to be optimized management with effectively utilizing the precipitation.

## 5 Conclusion

Limited irrigation is an optimized approach for irrigation management and irrigation amount had a significant impact on wheat grain yield and WUE. The level and time of water application need more control in limited irrigation than full irrigation. The semi-arid regions (site-2 and 3) indicated that almost 50% less water was beneficial to increase the wheat grain yield and WUE. Whereas the wheat grain yield was different under fourteen irrigation treatments for arid and semi-arid regions. The actual irrigation level was suitable for achieving both high yield (2394 kg ha^−1^) and high WUE (5.9 kg^−3^) in the arid region. Overall T_1_, T_11_, and T_9_ treatments were considered the optimal irrigation levels for efficient high wheat grain yield production in arid and semi-arid sites respectively. In conclusion of the research findings, it is recommended that water scarcity under arid climatic conditions should be prevented. Further research is needed to finish the recent and future deficit irrigation strategies for the study area and once optimum strategies are developed, field testing should be performed to verify them.

## Funding Information

Swedish University of Agricultural Sciences will grant the article publishing fee.

## Declaration of Competing Interest

The authors declare that they have no known competing financial interests or personal relationships that could have appeared to influence the work reported in this paper.
